# The chronic coronary syndrome—Heart failure roundabout: A multimodality imaging workflow approach

**DOI:** 10.3389/fcvm.2022.1019529

**Published:** 2022-11-22

**Authors:** Radu I. Lala, Simona Mercea, Radu A. Jipa, Maria Puschita, Adina Pop-Moldovan

**Affiliations:** ^1^Department of Cardiology, Arad Emergency Clinical County Hospital, Arad, Romania; ^2^Department of Cardiology, “Vasile Goldis” Western University of Arad, Arad, Romania

**Keywords:** chronic coronary syndrome, heart failure, stress, cardiac computed tomography angiography, cardiovascular magnetic resonance

## Abstract

Heart failure (HF) is a complex syndrome of considerable burden with high mortality and hospitalization rates. Approximately two-thirds of patients with HF have ischemic etiology, which makes crucial the identification of relevant coronary artery disease (CAD). Moreover, patients with chronic coronary syndrome (CCS) can first show signs of dyspnea and left ventricular (LV) dysfunction. If establishing a diagnosis of HF and consequent management is clear enough, it will not be the same when it comes to recommendations for etiology assessment. Ischemic heart disease is the most studied disease by cardiac multimodality imaging with excellent diagnostic performance. Based on this aspect, the high prevalence of CAD, the worst outcome—HF patients should undergo a diagnostic work-up using these multimodality imaging techniques. The aim of this mini-review is to provide insights on multimodality imaging for diagnosing CCS in patients with new onset of HF and propose a diagnostic work-up based on current international studies and guidelines.

## Introduction

Chronic Coronary Syndrome (CCS) is the leading cause of heart failure (HF) worldwide, especially in developed countries (1). Cardiac remodeling with consequent HF is not only seen after myocardial infarction but also in patients with CCS due to severe chronic ischemia.

The incidence of newly diagnosed HF among patients with chronic CCS ranges between 7 and 28% in reports from major registries (2). CCS is responsible for worsening HF and an increase in HF hospital admissions (3). The international CLARIFY study showed that after a 5-year follow-up, 16.4% of over 30,000 patients with baseline chronic coronary artery disease (CAD), developed a primary outcome of cardiovascular (CV) death, hospitalization for HF and new-onset of HF (4). Acute onset of HF carries a worse outcome, thus it is important to rapidly determine the etiology for accurate treatment. Because ischemic heart disease is the main driver of HF and because of its potential reversibility, a special focus should be addressed on diagnosing ischemia in all patients with new onset of HF of unknown cause.

The 2019 Guidelines on diagnosis and management of CCSs introduce six different clinical scenarios most frequently encountered in patients with stable CAD, all of which involve various risks for future cardiovascular events (5). The second scenario with the highest CV burden encountered is represented by patients with new onset of HF and left ventricular (LV) dysfunction and suspected CAD (5). Another update in the recent guidelines is the addition of dyspnea into the classic Diamond and Forrester classes of the pre-test probability of CAD (5). Dyspnea may accompany angina but may also be the sole symptom of coronary artery disease (CAD); sometimes it may be difficult to differentiate this aspect from other conditions. From a simple point of view, dyspnea in a patient with new onset of HF together with LV dysfunction substantially increases the clinical likelihood of CAD. Of course, one should consider the presence of other cofounders: family and clinical history, smoking, hypertension, diabetes, dyslipidemia, ECG, and echocardiographic abnormalities. Multimodality cardiac imaging [stress echo, coronary computed tomography angiography (CCTA), cardiovascular magnetic resonance (CMR), and single photon emission computed tomography (SPECT)] is at the core of diagnosing CAD. Based on the clinical likelihood of CAD and patient characteristics, non-invasive functional imaging for myocardial ischemia (stress echo, stress CMR, and SPECT) or CCTA is recommended as the initial test to diagnose CAD in symptomatic patients for whom obstructive CAD is uncertain by clinical assessment alone (5). In our opinion, this is characteristic of a wide range of patients with HF. The 2021 Guidelines on acute and chronic HF regarding diagnostic work-up, recommends to rule out chronic CAD, by taking into consideration CCTA in those patients with low to intermediate pre-test probability and equivocal non-invasive stress tests, while CMR should be considered in those patients with dilated cardiomyopathy to distinguish between ischemic and non-ischemic cardiomyopathy (6).

Based on current guidelines and recent evidence, a diagnostic work-up algorithm is needed based on cardiac imaging modalities to rule out ischemic cardiomyopathy as a potential cause for new onset of HF of unknown cause. These cardiac imaging modalities are not only safe and non-invasive but provide valuable data regarding coronary anatomy, myocardial ischemia, and tissue characterization.

## Stress echocardiography

Stress echocardiography is a widely used technique recommended by the European Guidelines as an initial test in symptomatic patients for functional assessment of myocardial ischemia and for whom CAD cannot be excluded by clinical examination alone (5). In case of HF, stress echo should be performed in patients already known with CAD who are considered for revascularization in order to assess ischemia and viability (6). Stress echocardiography has an overall sensitivity and specificity for predicting the recovery of myocardial regional function by 84 and 81% (7). Viable myocardium is a rather complex term because, from a molecular point of view, it can be described by different mechanisms such as intact cell membrane, residual glucose metabolism and contractile reserve. This particularity may explain the differences in the sensitivity and specificity of imaging techniques. For example, Bax et al. showed a higher specificity of echo-dobutamine stress test compared to positron emission tomography (PET) or SPECT as there is a possibility that in hibernating myocardium contractile reserve may be lost, but with preserved basal glucose metabolism and cell membrane integrity (8). Patient conformation (obesity, emphysema, and thoracic deformations—poor acoustic window), operator experience dependency and respiration artifacts limits the use of this technique to a first step or connection road with more complex imaging techniques like CMR or cardiac CT.

**FIGURE 1:**
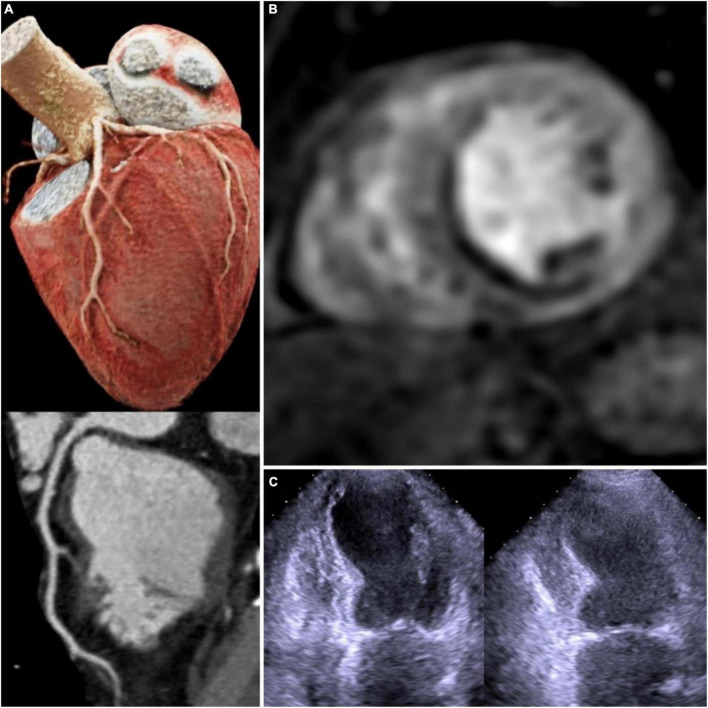
Multimodality imaging in chronic coronary syndrome: 3D CT coronary angiography with left anterior descendent artery curved-MPR–no lesions **(A)**; stress CMR with adenosine—subendocardial infero-septal perfusion deficit **(B)**; dobutamine stress echocardiography—apical inferior wall hypokinesia **(C)**. 3D CT, 3 dimensional computed tomography; MPR, multiplanar reformation; CMR, cardiovascular magnetic resonance.

Overall, due to its wide availability, low cost, lack of ionizing radiation, supporting evidence, stress echocardiography remains an attractive first-choice imaging technique in patients with ischemic cardiomyopathy (Figure 1C).

## Cardiovascular magnetic resonance

The presence of CAD in patients with HF and reduced ejection fraction (EF) is predictive of long-term mortality (9). It is rather challenging to detect CAD accurately in patients with HF, which is why there is a need for a standard imaging technique that can overcome the technical deficiencies of the traditional cardiac imaging methods. Due to its excellent spatial resolution, CMR seems to be the best candidate for this matter. CMR with late gadolinium enhancement (LGE) imaging can accurately distinguish between ischemic and non-ischemic cardiomyopathy by characterizing the presence, the localization, and the extent of the myocardial scar. In one study, CMR has shown a diagnostic accuracy for discerning ischemic etiology in patients with new onset of HF, higher than 95%, similar to that of coronary angiography (10). Acquiring LGE images is necessary for a stress CMR because it can differentiate between perfusion defects caused by myocardial ischemia or fibrosis. The absence of LGE with the presence of perfusion defects defines inducible ischemia (11) (Figure 1B). The presence of LGE is paramount in HF patients because it provides information about myocardial viability. Subendocardial scar with different grades of transmural extent was shown to correlate with the likelihood of functional recovery after revascularization (12). For example, a subendocardial scar with < 50% transmurality is considered to be viable myocardium especially in patients with old myocardial infarction or chronic total occlusion coronary lesions (12, 13). The presence of LGE carries a poor outcome prognosis in patients with CAD, especially in those patients with low EF; furthermore it can guide defibrillator implantation or resynchronization therapy (14).

Stress CMR is a validated functional imaging test for myocardial ischemia detection. It holds a valuable importance because it offers a multiparametric approach: cardiac function evaluation, myocardial scar detection, and dynamic reproducible ischemia. Stress CMR has been shown to have excellent diagnostic accuracy for detecting CAD in either the case of critical coronary stenosis or microvascular dysfunction (15). In 2001, stress CMR came for the first time into the spotlight, when Schwitter et al. demonstrated in an unselected study population that the efficiency of this technique in detection of CAD (PET scan and coronary angiography were used as the gold standard for diagnosis) has a sensibility and specificity ranging from 87 to 94% (16). Later, three multicenter prospective trials MR-IMPACT I, MR-IMPACT II, and CE-MARC showed the power of stress CMR for CAD detection in comparison with SPECT (17–19). Basically, both MR-IMPACT and MR-IMPACT II pointed out that stress CMR had a superior diagnostic performance and sensitivity compared to SPECT for CAD detection with invasive coronary angiography (ICA) as reference standard (AUC 0.67, *p* = 0.013; AUC 0.75 vs. 0.65, *p* = 0.0004) (17, 18). In the CE-MARC study, stress CMR was proven to have a significantly higher sensitivity with negative predictive value (86 vs. 66%, 90 vs. 79%, *p* < 0.0001) and similar specificity with positive predictive value (83 vs. 82%, 77 vs. 71%, *p* = 0.061) in comparison to SPECT (19). On top of CE-MARC later came CE-MARC II trial which showed a higher efficiency of CMR guided care in comparison to National Institute for Health and Care Excellence (NICE) guidelines for unnecessary ICA for patients with suspected CAD (20).

The diagnostic performance of stress CMR was tested by Pontone et al. in a meta-analysis of 77 studies on CCS where invasive fractional flow reserve (FFR) was considered the gold standard for diagnosing CAD. The study demonstrated higher sensitivity of stress CMR (81%) in detecting functionally significant coronary artery stenosis when compared to stress perfusion CT+CCTA (79%), stress echo (72%), SPECT (64%) but with lower sensitivity to FFR-CT (85%), CCTA alone (88%), and PET (87%) (21). In the studies mentioned above, ischemia was evaluated by visual assessment of myocardial segments perfusion deficits under adenosine stress. Besides visual assessment of myocardial perfusion deficits, there are also semi-quantitative and fully quantitative methods to quantify myocardial blood flow at rest and stress. These new methods, although not yet clinically validated, are based on the evaluation of signal intensity time curves and have recently gained attention due to their high sensitivity and specificity for assessing coronary microvascular dysfunction and myocardial perfusion reserve (22). This is of particular importance in HF patients as microvascular dysfunction is an essential pathophysiological mechanism for this population, especially for those with HF with preserved EF, myocardial infarction and non-obstructive coronary arteries (MINOCA) with LV dysfunction or diabetic cardiomyopathy. Mordini et al. demonstrated in a study on 67 patients with clinical suspicion of myocardial ischemia that fully quantitative stress perfusion CMR with dipyridamole has a high diagnostic accuracy for detecting obstructive CAD with a sensitivity of 87% and specificity of 93% (23). In another study, Levelt et al. demonstrated microvascular dysfunction by stress CMR in patients with diabetes without significant CAD (24). One study even pointed out a good correlation between myocardial blood flow and perfusion reserve assessed by stress CMR and invasive measurements (25).

All in all, stress CMR has gained a well-deserved place in the clinical guidelines and is now considered a trustworthy technique for myocardial ischemia assessment. However, there are some main limitations of stress CMR, and these are: limited spatial coverage, susceptibility artifacts in patients with cardiac devices, patients with claustrophobia, higher acquisition times and limited coronary anatomy analysis due to reduced spatial resolution (26, 27). Because of its low cost, lack of ionizing radiation, emerging quantitative perfusion techniques, and multiparametric approach, it is more likely that soon stress CMR will gain even more important attributions for diagnosing and managing CAD.

## Coronary computed tomography angiography

Coronary computed tomography angiography is recommended as a first-choice option for diagnosing CAD where there is clinical suspicion with low-probability according to stable CAD guidelines and should be considered in HF patients with low-intermediate likelihood of CAD according to HF guidelines (5, 6). This is a rather challenging task considering symptom variability and clinical cofounders in the HF population. However, CCTA presents a high likelihood to “rule out” and a low likelihood to “rule in” CAD (5, 28). Despite its ionizing radiation and possible contrast toxicity with the development of novel prospective gating techniques that require much lower dose radiation, and because of a shorter scan time, CCTA is an attractive option to CMR. It can accurately evaluate coronary anatomy, which is very helpful in HF patients when ruling out CAD as a possible cause (Figure 1A). In the evaluation of integrated cardiac imaging for the detection and characterization of ischaemic heart disease (EVINCI) study, CCTA had better diagnostic accuracy than stress CMR, PET, SPECT, or stress echocardiography for diagnosing ischemic heart disease (29). Im et al. in a prospective study, evaluated the etiology of new onset HF by using CCTA for coronary anatomy assessment and delayed-enhanced dual energy computed tomography for myocardial fibrosis assessment. Ischemic HF was diagnosed in 30% of patients while non-ischemic HF in 70% and the concordance between cardiac CT and clinical decision was 92%. In a meta-analysis by (30) CCTA was shown to have excellent diagnostic accuracy in diagnosing significant CAD in patients with new onset of HF (31).

Coronary computed tomography angiography can qualitatively and quantitatively assess coronary stenoses and provide valuable information on plaque features more accurately than other imaging techniques (11). These features include: spotty calcifications, fibrous cap thickness, low-attenuation plaque, positive remodeling index, which are very useful, especially when dealing with a high risk plaque (32). In the case of plaques with a high calcium burden due to “blooming” artifacts it is very difficult to quantify stenosis severity by CCTA. This together with several other limiting factors gives less specificity to CCTA. Considering this, new CT methods have emerged for additional functional non-invasive testing of myocardial ischemia. Although not recommended in the guidelines, FFR-CT and stress computed tomography perfusion (CTP) showed promising results in identifying critical coronary lesions and inducible ischemia.

FFR-CT is the non-invasive correspondent of FFR ICA and is defined by the ratio of flow distal and proximal to stenosis at rest or under stress conditions by computational fluid dynamics (33). The diagnostic performance of FFR-CT was tested in DISCOVER-FLOW and the NXT Trial in comparison to invasive ICA-FFR with a sensitivity of 86% and specificity of 79% (34, 35). One of the largest trials to test FFR-CT on more than 5,000 patients with suspicion of ischemic heart disease was the ADVANCE study which showed that a negative FFR-CT (> 0.80) predicted lower major adverse cardiovascular events and less revascularization procedures (36). The RIPCORD study showed that adding FFR-CT to CCTA could change treatment strategy (optimal medical treatment or invasive procedures). For example, 29% of coronary stenoses that were considered critical anatomical lesions by CCTA were proven to actually have a normal FFR (37). Stress CTP comes in addition to CCTA for functional assessment of anatomical coronary lesions by inducing ischemia under a stress agent such as adenosine or regadenoson. In the PERFECTION study, Pontone et al. compared the diagnostic accuracy and efficacy of static stress CTP to CCTA or FFR-CT on patients suspected of CAD. The study showed a higher diagnostic performance of static stress CTP in addition to CCTA than CCTA alone and the same diagnostic performance as FFR-CT+CCTA (38). Dynamic stress CTP in a meta-analysis where invasive-FFR was considered the reference method, proved to have a sensitivity and specificity of 85 and 93% with positive predictive value for CAD (39).

The main advantage of CCTA is the possibility to evaluate in the same examination with high precision coronary stenosis while providing functional assessment for ischemia by adding stress perfusion or FFR-CT analysis. However, the main drawbacks are reduced temporal resolution and contrast-to-noise ratio, the use of ionizing radiation and iodinated contrast media with possible nephrotoxicity in patients with impaired renal function (40).

## Nuclear cardiac imaging

### Single photon emission computed tomography

Untill the appearance of stress CMR and stress CT, SPECT myocardial perfusion imaging (MPI) was the pillar in assessing functional ischemia in patients with suspicion of CAD. SPECT utilizes Tc99m radiotracer for the evaluation of blood flow distribution during stress and rest and also the cell membrane integrity. The radiotracer can identify transient regional myocardial perfusion deficit which is indicative of ischemia while on the other hand a fixed perfusion defect reflects myocardial scar or fibrosis. Underwood et al. showed in a meta-analysis that SPECT reached a sensitivity of 87% and specificity of 73% in diagnosing significant CAD (41). Even tough radionuclide MPI enjoys a long-term experience and validation studies for CAD diagnosis including risk stratification it holds a rather insensitive accuracy when it comes to diffuse obstructive atherosclerosis and microvascular dysfunction (42).

### Positron emission tomography

In PET scan, the radiotracers used (rubidium, N-ammonia) possess superior extraction characteristics and shorter half-lives which makes them more adequate for myocardial blood flow quantification while facilitating lower radiation doses (43). In the PACIFIC trial, PET showed a higher sensitivity (> 97%) than CCTA for ruling out high-risk obstructive CAD (44). PET with F-FDG tracer can be used to assess myocardial glucose metabolism and viability which is highly important in the management of HF patients. The advantages of PET are: high spatial and contrast resolution, dynamic imaging with high temporal resolution, CT hybrid imaging and low radiation dose protocols (45). PET offers valuable data regarding ventricular function, myocardial perfusion, myocardial metabolism, viability and intraventricular synchronism, thus providing high quality imaging for quantitative analysis which makes it appealing for assessing HF patients (46). However, due to less availability in some centers, lower spatial resolution in comparison to CMR, higher costs, radiation exposure, lack of anatomical assessment of coronary plaque burden and scientific data variation regarding diagnostic performance, the role of PET for HF etiology assessment is not a first choice and should be limited to specific recommendations and center experience.

## Is there a winner?

When it comes to properly assessing HF etiology the answer is “no.” One should keep in mind that a newly diagnosed patient with HF needs a comprehensive anatomical, functional, and viability assessment. It is ideal to have one imaging technique that comprises all the aforementioned features. Of course, there are imaging techniques that can assess all, but as shown in studies, cardiac imaging techniques have different sensitivities and specificities, which is why a multimodality imaging approach is needed. The question is: which is the better approach? Clearly, for anatomical coronary assessment, CCTA is the first and most accurate choice. For the functional assessment of ischemia, both stress CMR and stress CTP integrated to computed tomography angiography (CTA) have similar diagnostic accuracies (47). However, it is still important to have knowledge of the coronary anatomy for future revascularization techniques. For example, the recent SYNTAX III Revolution study conducted by Andreini et al. demonstrated that FFR-CT with CCTA alone can guide out the type of revascularization technique even better than ICA (48).

**FIGURE 2:**
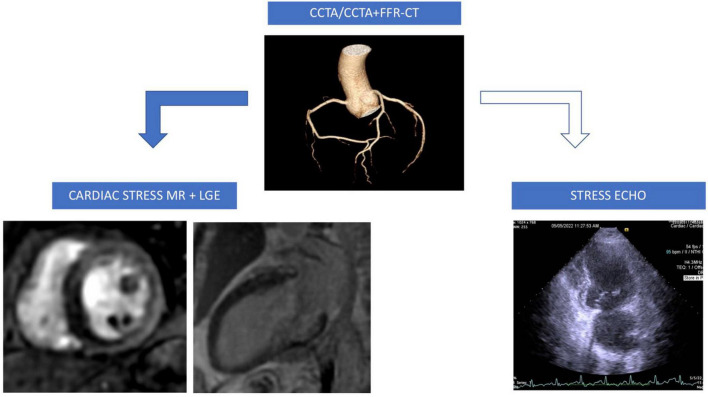
A proposed clinical approach algorithm for ischemic cardiomyopathy diagnosis: If coronary lessions (intermediate lessions 50–75% stenoses) are detected by CCTA then perform FFR-CT if available (in experienced centers) otherwise perform stress CMR with LGE (transmural infero-septal perfusion deficit, basal inferior wall subendocardial scar); if FFR-CT and stress CMR not available then perform stress echocardiography. CCTA, coronary computed tomography angiography; FFR-CT, fractional flow reserve computed tomography; CMR, cardiovascular magnetic resonance; LGE, late gadolinium enhancement.

Baggiano et al. proposed a multimodality algorithm approach for clinical suspicion of CAD (49). As a first step, all patients should undergo CCTA. If coronary stenoses > 50% are detected not involving left main or proximal left anterior descending artery, then the next step would be to functionally assess myocardial ischemia by FFR-CT/stress CTP depending on the local expertise and availability. If neither technique is available, then stress echo or stress CMR would be the next choice (49). Although highly relevant, when it comes to HF, especially with reduced EF, it is crucial to know myocardial viability and tissue characterization of possible non-ischemic etiologies. For this, CMR is the number one choice due to its high spatial resolution, diagnostic and prognostic role, although CT with delayed enhancement for tissue characterization has gained recent attention but at the expense of lower spatial resolution. Thus, in patients with new-onset and established diagnosis of HF, we propose the following algorithm (Figure 2): a first step would be CCTA for rapid exclusion of CAD; if stenoses > 50% are present, the next step would be to perform a stress CMR with LGE characterization. If CMR is not available, we propose, as a reasonable choice, a dobutamine stress echo. In advanced centers, if available, we suggest an FFR-based CT approach on top of CCTA if > 50% stenoses are present, followed by CMR with LGE tissue characterization. No matter what imaging tests are chosen, or no matter their availability, or which one fits better the patient’s pathology, it seems to be certain that CCTA in both CCS and HF is the cornerstone on which diagnosis and management will be further built.

## Conclusion

Cardiac multimodality imaging is a continuously growing discipline that seems to have a pivotal role in different cardiac pathologies. Time is not on the patient’s side from the first moment the diagnosis of HF is made, therefore a proper and precise workflow assessment of etiology should be done as soon as possible to improve the prognosis. It is crucial to immediately exclude CAD as it is the most frequent etiology encountered in HF patients. To do so, a multimodality approach is recommended firstly by evaluating coronary anatomy by CCTA followed by functional and myocardial viability assessment by CMR.

## Author contributions

All authors contributed to the writing and revision of the manuscript and approved the final manuscript.
